# An LSPR-Active AuNP–Silicone Hydrogel Contact Lens for Continuous Ocular Strain Sensing: From Engineering Design to In Vivo Validation

**DOI:** 10.3390/bios16050296

**Published:** 2026-05-20

**Authors:** Yu Tang, Luhua Meng, Yun Liu, Xiang Ma

**Affiliations:** 1 Department of Ophthalmology, The First Affiliated Hospital of Dalian Medical University, Dalian 116011, China; tangyu0961@163.com; 2 Department of Ophthalmology, Central Hospital of Dalian University of Technology, Dalian 116033, China; 3 School of Physics, Dalian University of Technology, Dalian 116024, China; mlh871783468@163.com

**Keywords:** localized surface plasmon resonance, gold nanoparticles, silicone hydrogel, smart contact lenses, intraocular pressure, biocompatibility, wearable diagnostics

## Abstract

Continuous intraocular pressure (IOP) monitoring is crucial for glaucoma management. Currently, traditional static IOP measurements often fail to detect circadian fluctuations, leading to a clinical dilemma where “normal IOP” is observed despite persistent visual field deterioration. This study presents a wireless, passive localized surface plasmon resonance (LSPR) sensing platform integrated into flexible silicone hydrogel contact lenses. Gold nanoparticles (AuNPs), synthesized via the sodium citrate reduction method, were incorporated into the lens periphery using a “swelling-induced nano-doping” technique to transduce IOP-induced corneal strain into detectable spectral shifts. Ex vivo porcine eye investigations established a physical mapping model, confirming significant LSPR peak wavelength response trends in correlation with IOP variations (10–50 mmHg) and corneal curvature changes. Subsequent 21-day in vivo rabbit studies demonstrated excellent ocular surface biocompatibility; quantitative histopathological analysis (HE, PAS, and Ki67 staining) revealed no significant adverse alterations in corneal endothelial cell density or conjunctival goblet cell function compared to control groups (*p* > 0.05). Furthermore, the platform maintained high structural integrity and anterior segment tolerance under transient high-IOP conditions. While currently a proof-of-concept, these results indicate that the LSPR-active hybrid system effectively captures dynamic IOP fluctuation patterns as an optical response to acute interventions, providing a foundational engineering path for next-generation, battery-free wearable diagnostics in personalized glaucoma care without the need for built-in electronics.

## 1. Introduction

Glaucoma is a significant public health problem. Glaucoma is the second leading cause of blindness and the leading cause of irreversible blindness worldwide [1,2]. Glaucoma is a chronic, progressive ocular disease causing typical damage to the optic nerve head, the retinal nerve fibre layer, and the visual field [3]. It is estimated that by 2040 at least 112 million people will be diagnosed with glaucoma worldwide [4]. The measurement and dynamic monitoring of intraocular pressure (IOP) is an important basis for the diagnosis of glaucoma. IOP reduction is the primary strategy to treat glaucoma, and has been well established in clinical practice [5]. Long-term and effective monitoring and management of IOP can delay the loss of visual function, which has important clinical significance for the diagnosis and treatment of glaucoma [6]. Currently, Goldmann applanation tonometry (GAT) and non-contact tonometer (NCT) are both widely used methods for measuring IOP. But they are only single-point measurements and cannot capture the diurnal fluctuations of IOP, which may lead to missed diagnosis and inadequate treatment. Therefore, the development of non-invasive and continuous IOP monitoring technology has become an urgent need and research frontier in the field of ophthalmology.

With the continuous advancement of modern technology, wearable IOP monitoring technology, especially intelligent sensing corneal contact lenses, is an important ophthalmic medical device, the functions and application scope of which are constantly expanding. Smart contact lenses have evolved from simple corrective tools into transformative platforms for continuous health monitoring and personalized therapy [7,8]. The principle of using contact lenses to monitor IOP is based on Lam et al.’s study of corneal deformation characteristics: for every 1 mmHg change in IOP; the corneal curvature radius changes by 3 μm (when the corneal curvature radius is 7.80 mm) [9,10]. Flexible corneal contact lenses measure changes in corneal curvature radius caused by fluctuations in IOP by embedding sensing circuits into them to form a sensing layer [11,12]. In recent years, research has explored sensors based on the principles of resistance or capacitance, which indirectly estimate IOP by sensing changes in corneal curvature [13,14]. However, these solutions often face challenges: they require complex built-in circuits and power supplies, which may result in high lens rigidity, discomfort during wear, poor biocompatibility, and high costs, seriously hindering their clinical translation and widespread application.

The sensing technology based on optical principles provides a new path to solve the above problems. Van Duyne et al. proposed using the localized surface plasmon resonance (LSPR) of metal nanoparticles to design biosensors [15,16], which are based on the characteristic that the LSPR extinction peak shifts with changes in the environment in which the metal particles are located. Gold nanoparticles (AuNPs) serve as excellent carriers for LSPR, and their resonance peak wavelength shifts sensitively with small external deformations or refractive index changes. Recent reviews on flexible optical sensing platforms confirm that the integration of nanostructured materials like AuNPs into hydrogel substrates remains a frontier strategy for enhancing sensitivity and stability in 2025 [17]. They can be read through external optical systems without the need for a built-in power supply, making them highly suitable for constructing passive biosensors [18]. Due to its optical and electrical properties, as well as good biocompatibility, ease of modification, and low toxicity [19,20], AuNPs have been widely used in fields such as bioelectrochemistry, biological detection, biological diagnosis, and biosensors [21]. The introduction of this technology provides new possibilities for the functional expansion of corneal contact lenses.

According to the close correlation between IOP and corneal curvature and the structural characteristics of the cornea and sclera, this study presents a non-invasive, wearable optical sensor designed for the continuous tracking of dynamic IOP fluctuations. The sensing mechanism relies on the LSPR effect of AuNPs uniformly embedded within a silicone hydrogel matrix [22].

## 2. Working Principle and Design of Corneal Contact Lenses

The LSPR effect arises from the collective oscillation of free electrons on the surface of noble metal nanoparticles when excited by an incident light field [23]. These nanoparticles exhibit intense extinction cross-sections at specific resonant wavelengths, resulting in distinct extinction spectral bands within the ultraviolet–visible region. For AuNPs with diameters typically ranging from 1 to 100 nm, their extinction characteristics—including peak wavelength and spectral profile—are highly sensitive functions of particle size, morphology, and the local dielectric environment. Specifically, the extinction spectrum of an AuNP-embedded matrix is modulated by the spatial distribution of the nanoparticles. A decrease in particle distance enhances the plasmonic coupling effect, typically inducing a redshift and broadening of the extinction peak; conversely, increasing the spacing weakens this interaction, leading to a blue shift [24]. By monitoring these spectral extinction shifts in real time, the microscopic strain state of the host medium, such as a silicone hydrogel film, can be precisely quantified.

**Figure 1:**
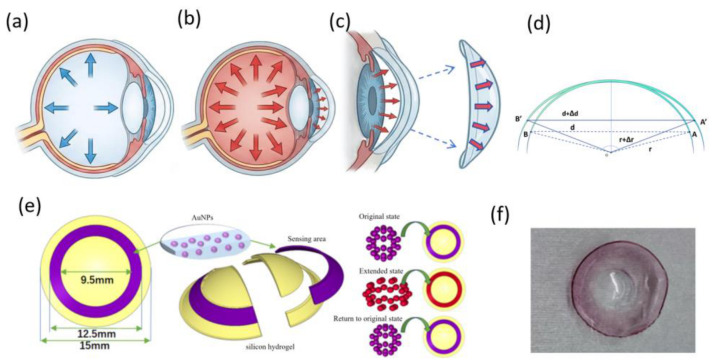
Design of AuNP–silicone hydrogel contact lens. (**a**) Normal IOP maintains the normal shape of the eyeball. (**b**) Elevated IOP causes eyeball expansion and changes in corneal curvature. (**c**) Changes in corneal curvature lead to alterations in the morphology of contact lenses covering the corneal surface. (**d**) A schematic diagram of the deformation of the contact lens induced by IOP change. (**e**) Sensing sites with metal nanoparticles are concentrically positioned at an 11 mm diameter, corresponding to the corneoscleral junction. (**f**) AuNP–silicone hydrogel contact lens.

As illustrated in Figure 1, elevations in IOP drive corneal expansion, consequently increasing the radius of curvature. Consider two representative points, A and B, on a corneal cross-sectional plane; as IOP rises, these points translate to A′ and B′. This geometric expansion increases the cross-sectional diameter from d to d + Δd and the circumference from πd to π(d + Δd), thereby extending the arc distance between the points. This macroscopic corneal deformation induces elastic strain within the silicone hydrogel film, effectively modulating the interparticle spacing of the embedded AuNPs. Leveraging the LSPR coupling effect, these spatial displacements are transduced into detectable spectral peak shifts. Consequently, the microscopic tensile deformation of the hydrogel matrix—and by extension, the IOP—can be directly inferred by analyzing the drift of the spectral extinction peak. To optimize sensitivity, the sensing units are strategically localized at the corneoscleral junction (≈11 mm diameter). This region was selected based the published results of corneoscleral biomechanical finite element analysis, which identifies it as the site of maximum local strain anisotropy during IOP fluctuations [25]. This composite architecture aligns with wearable optical biosensors: the hydrogel matrix ensures adequate breathability and mechanical compliance, while the integration of AuNPs has been shown to enhance oxygen permeability and minimize protein foulant adsorption, satisfying the rigorous requirements for long-term ocular wear [26].

AuNPs were synthesized using the sodium citrate reduction method. To obtain a corneal contact lens substrate material that possesses optical transparency, aqueous properties, and adaptability for subsequent nano-functionalization, a silicone hydrogel polymerization precursor system was first constructed. A monomer mixture system was prepared by mixing γ-hydroxyethyl methacrylate (HEMA), N-vinyl pyrrolidone (NVP), and methacryloyloxypropyl trimethoxysilane (KH-570) in a ratio of 8:1:1. Azobisisobutyronitrile (AIBN) was added as an initiator, and N,N′-methylenebis(acrylamide) (NMBA) as a crosslinking agent. After stirring at 1500 rpm for 30 min, undissolved impurities were removed through membrane filtration to obtain a uniform and transparent prepolymer solution. Among them, HEMA served as the main framework of the hydrophilic polymer network, NVP was used to improve the wettability and aqueous properties of the system, and KH-570 enhanced the flexibility and structural stability of the material by introducing silicone components, while the synergistic effect of AIBN and NMBA ensured the smooth progression of subsequent thermal polymerization reactions and the effective construction of the crosslinked network. Subsequently, the obtained prepolymer solution was injected into pre-cleaned contact lens molds and underwent staged thermal polymerization at 65 °C and 75 °C, enabling the system to complete initial gelation and further crosslinking solidification, ultimately obtaining a silicone hydrogel substrate lens with a stable three-dimensional network structure. The silicone hydrogel matrix was thermally cured and demolded to obtain the base hydrogel films.

To ensure an unobstructed field of view during wear, a localized “swelling-induced nano-doping” strategy was employed to functionalize only the lens periphery (3 mm wide) while maintaining a transparent central optical zone. The lenses were fully immersed in ultrapure water to remove unreacted monomers and small molecular residues, and to complete network hydration and structural equilibrium. The demolded lens was mounted onto a customized magnetic curved carrier that provided physical shielding for the central area. The entire assembly was immersed in acetone for 2 min to induce hydrogel network swelling and generate transient nanopores, followed by a 30 min immersion in an AuNP dispersion to allow for nanoparticle infiltration via diffusion. To ensure optimal doping uniformity and structural stability, this doping cycle was repeated 5–6 times. The resulting composite lens features a distinct architecture with a functionalized sensing edge and a clear center. While maintaining the fundamental optical transmittance, flexibility, and mechanical integrity of the lenses, localized surface plasmon characteristics are imparted to the material, providing a foundation for subsequent spectral response modulation and signal readout [27,28].

The corneal contact lenses used for ex vivo pig eyes have a base arc of 11.46 mm, a diameter 19.4 mm, and a thickness of 0.2 mm, with gold nanoparticles measuring 30 nm in diameter. The corneal contact lenses used for rabbit have a base arc of 8.4 mm, a diameter 13.8 mm, and a thickness of 0.04 mm, with gold nanoparticles measuring 50 nm in diameter.

**Figure 2:**
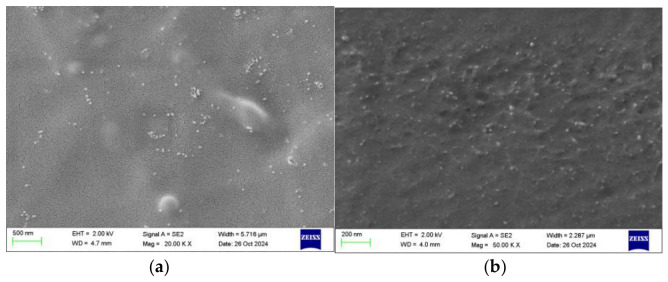
Scanning electron microscopy images of gold nanoparticles embedded in the surface (**a**) and cross-section (**b**) of silica hydrogel material.

**Figure 3:**
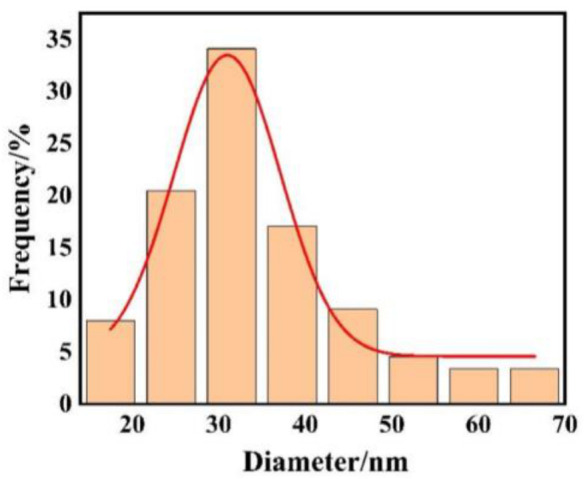
Size distribution profile of synthesized gold nanoparticles.

After embedding AuNPs into the silicone hydrogel matrix, we characterized the particle distribution using scanning electron microscopy (SEM) (Figure 2). Quantitative analysis of SEM images revealed an average surface density of approximately 5–8 particles/µm^2^ within representative surface observation areas (approximately 24.5 µm^2^). The prepared AuNPs exhibited an overall near-spherical morphology, but the particle size distribution was not entirely uniform, with most particles concentrated within the range of 30–45 nm, alongside a small number of larger-sized particles (Figure 3). The nanoparticles exhibited homogeneous dispersion with no significant large-scale agglomeration or macroscopic aggregation structures observed. In localized dense regions, particle spacing was markedly smaller than 200 nm, with some areas reaching tens of nanometers in size. The optical response of the system primarily originates from contributions of these localized high-density regions.

## 3. Experimental Method

### 3.1. Experimental Study on Fresh Ex Vivo Pig Eyes

Fresh detached pig eyeballs were stored in PBS buffer at 4 °C and transferred to the laboratory within 2 h. The eyeballs were fixed and a catheter was inserted through the anterior chamber. A pressure device and pressure tube were connected to the right side, and an optical fiber was placed above. A data acquisition and spectral detection system was placed on the left side. The fiber optic spectrometer (Ocean Optics, HR4000, China) was used for spectral acquisition. The instrument’s integration time could be set within the range of 3.8 ms to 10 s, with experimental parameters optimized based on detected signal intensity. Data collection frequency was set at one spectral data point every 0.2 s. The distance between the fiber probe and corneal surface was fixed at 0.8 cm (±0.05 cm). The pressure device was used to raise and lower the IOP. IOP was monitored using an Icare tonometer, while a handheld keratometer concurrently measured the corneal curvature radius. The peak light extinction of the contact lenses was characterized across a range of IOP levels and corresponding corneal curvatures, followed by a systematic spectral analysis to evaluate the optical response.

### 3.2. Experimental Study on Live Rabbit Eyes

**Figure 4:**
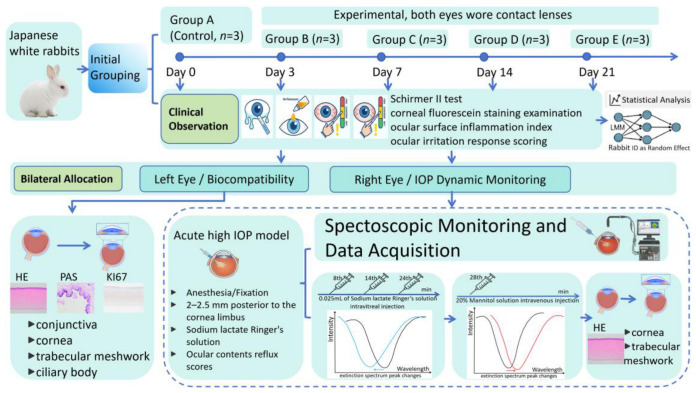
Experimental study on live rabbit eyes.

The experiment followed the statement of the Society for Visual and Ophthalmological Research on animal experiments and was approved by the Medical Ethics Committee (Approval Number: DUTSP250619-02). As shown in Figure 4, there were a total of 15 male Japanese white rabbits of ordinary grade, randomly divided into groups A–E (*n* = 3 rabbits per group) using a random number table method. Routine external eye examination was performed before the experiment to rule out eye diseases, and tear secretion test and corneal fluorescein staining were performed. Group A was the control group. Groups B–E were the experimental groups; both eyes wore contact lenses. Routine feeding, daily observation and recording of the general condition of experimental rabbits, and observation of conjunctival sac secretion, corneal contact lens position, etc., were carried out.

On the 3rd, 7th, 14th, and 21st day after wearing the corneal contact lenses, the lenses were sequentially removed from the B–E groups for the tear secretion test (Schirmer II test) and corneal fluorescein staining examination and scoring [29]. The experimental rabbits were evaluated and recorded according to the ocular surface inflammation index [30,31] and ocular irritation response scoring criteria [32]. Regarding histopathological evaluation, the left eyes were allocated for static wear assessment, whereas the right eyes were dedicated to post-IOP fluctuation analysis. This stratified design ensured a comprehensive evaluation of both long-term biocompatibility and functional sensing stability.

After collecting evaluation data, IOP fluctuation monitoring experiments were performed on each group of experimental rabbits (right eye). An acute high-IOP model was used by the vitreous cavity injection method. The rabbits were anesthetized with 20% urethane solution 5 mL/kg by intravenous injection at the ear margin. After anesthesia, the animals were fixed in the left lateral position, and the right eye was fitted with an optical fiber and data acquisition and processing system. The spectrum was continuously collected during the experiment. Sodium lactate Ringer’s solution (0.025 mL) was injected into the vitreous cavity in multiple doses at the posterior 2–2.5 mm to the corneal limbus, with injection times recorded and injection sites avoided to prevent repetition. During needle removal, ocular content reflux scores [33] were observed and documented. After completing injections and confirming stable spectral extinction peak recovery, 20 mL of 20% mannitol solution was rapidly administered via the marginal ear vein, with spectral extinction peak monitoring conducted within 5 min. After the completion of data collection, the rabbits in this group were euthanized by air embolism. The eyeball was completely removed, and the conjunctiva, cornea, trabecular meshwork, and ciliary body tissues were used for HE staining, PAS staining of the conjunctiva, KI67 immunohistochemical staining of the corneal tissue, and microscopic examination. Corneal endothelial cell counts were measured using Image-Pro Plus 6.0 software under low magnification for HE-stained sections. Nasal bulbar conjunctival cells were manually counted in 10 non-repetitive fields under high magnification after PAS staining, with boundary-crossing cells counted using the “left-upper” principle. All counts were performed by a single observer under a single-blind method.

SPSS 27.0 statistical software was used to analyze the data, and differences were considered statistically significant when *p* < 0.05. To address the inter-eye correlation within the same animal, a linear mixed-effects model was employed, treating the rabbit ID as a random effect. For single-eye data (e.g., histopathology), a one-way ANOVA with Tukey’s post hoc test was utilized. After the spectral acquisition experiment, Origin 2021 software was used for subsequent analysis. The raw temporal data points were determined by extracting the absolute peak wavelength of the LSPR extinction band at each time point. All fitting parameters were determined automatically by the algorithm without manual constraints on the peak/dip positions.

## 4. Results

### 4.1. Experimental Results of Fresh Ex Vivo Pig Eyes

**Figure 5:**
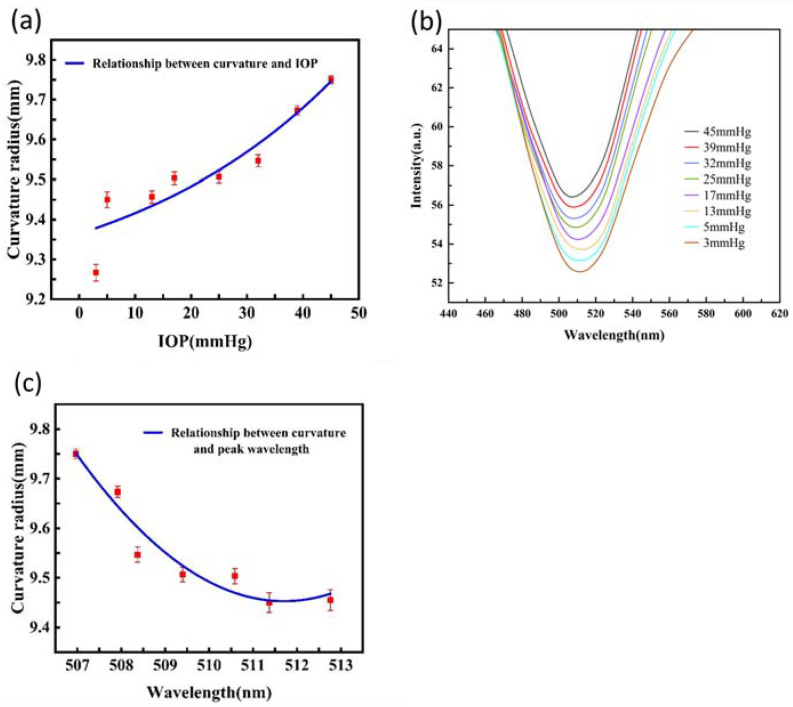
The relationship between IOP, corneal curvature radius, and peak wavelength of contact lens extinction spectra in ex vivo pig eye experiments. (**a**) The relationship and fitting curve between IOP and the corneal curvature radius. (**b**) The relationship between IOP and the peak wavelength of the contact lens extinction spectrum. (**c**) The relationship and fitting curve between the peak wavelength of contact lens extinction spectrum and the corneal curvature radius. The data points represent the average values of repeated measurements on the same eye sample to demonstrate the precision of the sensor.

As illustrated in Figure 5, the shift in the LSPR extinction peak of the smart contact lens demonstrates a strong correlation with IOP fluctuations in fresh ex vivo porcine eyes. An inverse relationship is observed: as IOP increases, the peak wavelength blue-shifts due to the concurrent increase in the corneal curvature radius. Since each IOP value corresponds to a specific corneal geometry for a given eye, a physical mapping model was established by correlating the spectral signals with these curvature variations. Linear regression analysis of the experimental data reveals a response sensitivity (slope S) of −0.12 nm/mmHg with a correlation coefficient (R^2^) of 0.859. This sensing platform leverages the stable optical properties of the lens material, facilitating functional optical responsiveness for non-invasive, dynamic IOP tracking.

### 4.2. Experimental Results of Live Rabbit Eyes

During the 21-day in vivo evaluation, the physiological performance of the smart contact lenses was closely monitored. The lenses exhibited a high centration rate, maintaining a stable position centered on the cornea with a typical post-blink movement, which facilitated adequate tear turnover. Importantly, the central optical zone remained clear and free of significant mucoid deposits or protein opacification throughout the experiment, ensuring that the visual axis remained unobstructed.

The experimental rabbits were generally in good condition, with tear secretion tests (Schirmer II test) all > 5 mm. The corneal fluorescein staining scores (expressed as mean ± standard deviation) for the different groups. The scores were as follows: Group A (0.08 ± 0.20), Group B (0.17 ± 0.26), Group C (0.25 ± 0.27), Group D (0.25 ± 0.41), and Group E (0.41 ± 0.49). A one-way analysis of variance (ANOVA) was conducted to compare the scores among the groups, resulting in an F-statistic of 0.764 and a *p*-value of 0.559. There were no statistically significant differences in corneal fluorescein staining scores among the groups under study. The evaluation of ocular surface inflammation index only showed mild central corneal edema in the right eye of experimental rabbit D3, but the iris was visible. That score was 1 point, and the rest were 0 points. The grading criteria for eye irritation response were as follows: all experimental rabbits were classified as non irritating (score 0–3), with a score of 1 for mild conjunctival congestion in the left eye of C2 and D3, a score of 2 for mild conjunctival congestion in the right eye of E2, and a score of 0 for the rest.

**Figure 6:**
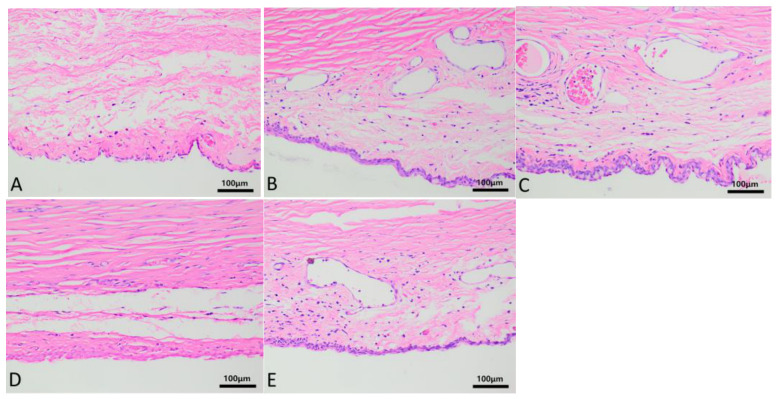
Detailed images of conjunctiva HE staining between (**A**–**E**) groups after prolonged continuous static wear of corneal contact lenses. (**A**) The conjunctival structure was normal with no obvious pathological changes. (**B**–**E**) The bulbar conjunctival blood vessels were mildly dilated with a small amount of inflammatory cell infiltration, without significant edema or fibrosis. In all groups, non-keratinized squamous columnar epithelium was closely arranged, goblet cells were scattered, and the lamina propria contained capillaries and lymphocytes with intact structure.

**Figure 7:**
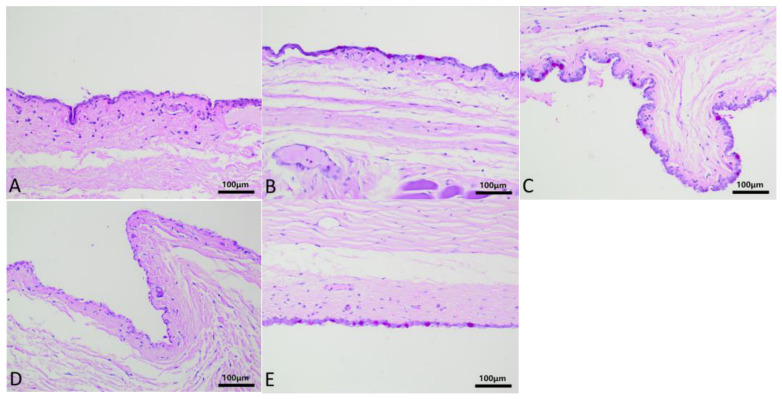
Detailed images of conjunctiva PAS staining between (**A**–**E**) groups after prolonged continuous static wear of corneal contact lenses. Scattered purple red particle clusters of goblet cells can be seen in the conjunctival epithelial layer of groups (**A**–**E**).

Group A–E rabbits wore corneal contact lenses continuously for a long time, and HE staining of the conjunctival tissue is shown in Figure 6. Non-keratinized squamous columnar epithelial cells are observed in the conjunctival tissue, with pale nuclear staining, regular and complete morphology, tight cell arrangement, and smooth epithelial surface. Basal cells are cuboidal with deeply stained nuclei. The lamina propria contains capillaries and diffuse lymphocytes, and no fibrosis is observed. After wearing contact lenses, mild dilation of the conjunctival blood vessels and infiltration of a small amount of inflammatory cells were observed in the B–E groups, and no significant conjunctival edema or thickening was observed. The PAS staining of the conjunctiva is shown in Figure 7. Under low magnification, scattered purple red particle clusters of goblet cells can be seen in the conjunctival epithelial layer of groups A–E. Under high magnification, the cytoplasm of goblet cells is filled with strong PAS+ substances, and the blue staining of the nucleus is biased towards the basal part. The typical high-footed cup shape is clearly visible. The epithelial basement membrane appears as a continuous and homogeneous purple red band, with no evidence of basement membrane rupture. The manual counts of goblet cells from PAS-stained conjunctival tissue sections for groups A to E are expressed as the mean number of cells ± standard deviation ($\bar{x}$ ± s). The counts were as follows: Group A (46.4 ± 3.85), Group B (48.6 ± 3.71), Group C (46.6 ± 4.33), Group D (44.4 ± 3.05), and Group E (45.2 ± 2.39). A one-way analysis of variance (ANOVA) was performed to compare the mean counts across the groups, which yielded an F-value of 1.021 and a *p*-value of 0.421. This result indicates that there were no statistically significant differences in goblet cell counts among the five groups.

**Figure 8:**
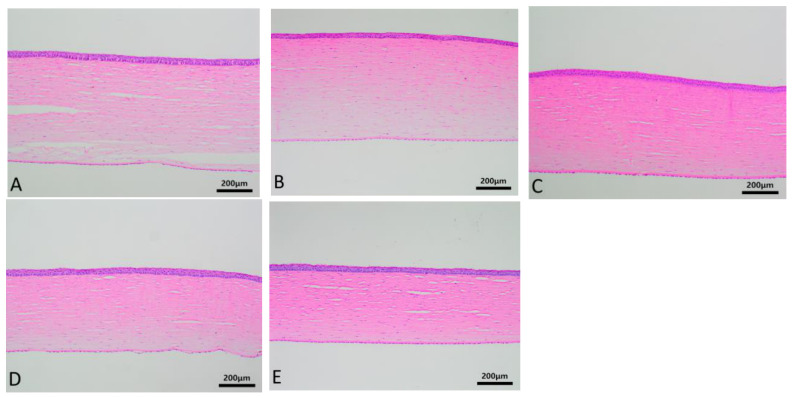
Detailed images of corneal HE staining between (**A**–**E**) groups after prolonged continuous static wear of corneal contact lenses. The epithelium is smooth and intact, with neatly arranged squamous epithelial and basal cells. The stromal collagen is regularly aligned, and the endothelium exhibits round, pale nuclei without evident edema, vacuolation, vascularization, or inflammatory cell infiltration.

**Figure 9:**
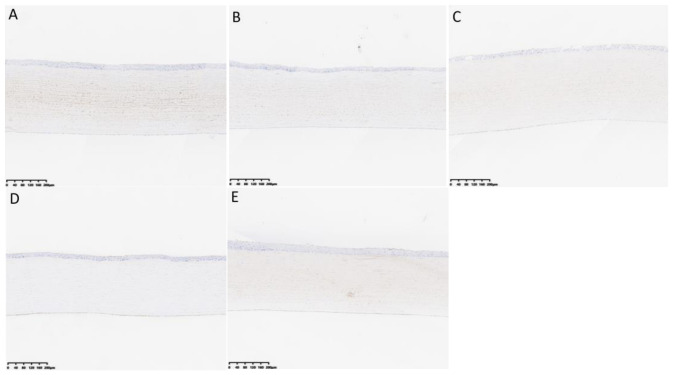
Detailed images of corneal KI67 staining between (**A**–**E**) groups after prolonged continuous static wear of corneal contact lenses. No positive staining was observed.

The HE staining of corneal tissues in each group is shown in Figure 8. Under low magnification, the corneal tissues are clearly layered, with smooth and intact epithelium, and the arrangement of squamous epithelial cells and basal cell layers is neat and uniform. The arrangement of collagen layers in the matrix layer is regular. Corneal endothelial cells have round pale nuclei with unclear cell boundaries, and no obvious edema or vacuolar changes are observed. The entire corneal tissue shows no infiltration of blood vessels, lymphatic vessels, or inflammatory cells. No positive staining was observed in any of the KI67 staining groups, as shown in Figure 9. The manual counts of corneal endothelial cells from hematoxylin and eosin (HE)-stained corneal tissue sections for groups A to E are presented as the mean number of cells ± standard deviation ($\bar{x}$ ± s). The cell counts for each group were as follows: Group A (43.53 ± 5.13), Group B (45.87 ± 3.70), Group C (43.53 ± 3.68), Group D (46.67 ± 4.67), and Group E (44.00 ± 4.24). Statistical analysis (one-way ANOVA) was performed to compare the counts across the groups, and no statistically significant differences were found (*p* > 0.05).

**Figure 10:**
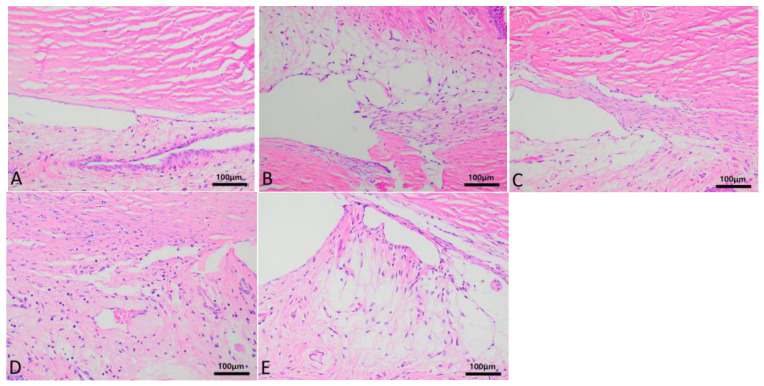
Detailed images of triangular trabecular meshwork HE staining between (**A**–**E**) groups after prolonged continuous static wear of corneal contact lenses. The morphology of trabecular cells is normal.

Figure 10 shows the triangular trabecular meshwork area at the corneal edge under low magnification, with regular arrangement of fibrous scaffolds in the form of purple red thick filamentous structures interwoven into a network. The morphology of trabecular cells is normal, and the nuclei are clearly visible. The pore structure inside the trabecular meshwork is clear, and no inflammatory cell infiltration, fibrosis, proliferation, or neovascularization is observed.

**Figure 11:**
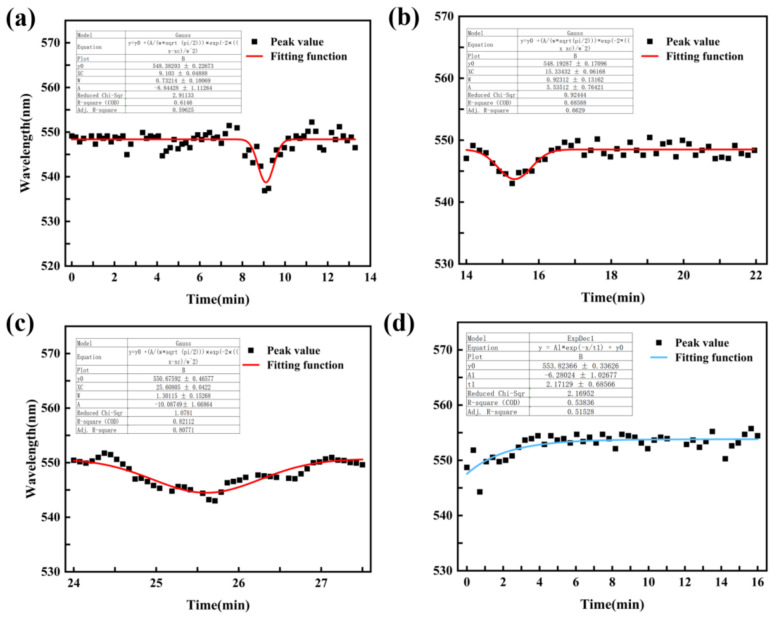
The extinction wavelength of corneal contact lenses in the fluctuating state of rabbit eye pressure intervention corresponded to the fluctuation change. The figure shows the raw peak extinction wavelength (black squares) and the corresponding non-linear fitting curves (solid lines) representing the dynamic LSPR shifts over the experimental time. (**a**) After the first vitreous cavity injection of sodium lactate Ringer’s solution, the spectral peak shifted to the left and quickly recovered; (**b**,**c**) After the subsequent two injections, the peak shift amplitude decreased and the recovery rate slowed down; (**d**) After intravenous injection of mannitol, the spectral peak gradually increased and tended to be stable.

On the day of static wearing, one rabbit from each group was randomly selected to conduct an eye pressure fluctuation monitoring experiment under intervention. The experiment ended within 1 h, and the eye content reflux score after intravitreal injection was less than 1 point. Figure 11 shows the changes in the peak extinction wavelength of the contact lens during one group of IOP intervention states. The figure shows the fitting curve between the peak extinction spectrum of the corneal contact lens and the experimental time. The sodium lactate Ringer’s solution was injected at the 8th, 14th, and 24th minutes. At the 28th minute, the timer was restarted and 20% mannitol solution was injected into the ear vein; 20 mL was rapidly injected within 3 min. Figure 11a shows that the baseline state is from 0 to 8 min, and the peak extinction spectrum of the corneal contact lens is stable at around 550 nm. At the 8th minute, 0.025 mL of sodium lactate Ringer’s solution was injected into the vitreous cavity, and the spectrum rapidly shifted to the left. Within 1 min, the spectral peak shifted to about 530 nm, and then shifted to the right for 1 min, quickly returning to the baseline state and maintaining stability. Figure 11b shows the injection of 0.025 mL again at the 14th minute, and the spectral peak shifts left to about 545 nm at 1 min, followed by a right shift of nearly 550 nm within 1 min. Figure 11c shows the injection of 0.025 mL again at the 24th minute, and the spectral peak shifts left to near 545 nm at about 1.5 min, with a slower rate of left shift compared to Figure 11b, and then slowly moves right to near 550 nm. During the process of reducing IOP, starting from the injection of 20% mannitol solution, the spectral wavelength peak gradually increases, the slope of the spectral wavelength peak change gradually decreases, the wavelength peak slowly reaches the plateau period, and the spectral peak remains stable. In the in vivo intervention experiment, IOP was modulated using intravitreal injections and mannitol administration to assess the sensor’s dynamic tracking capability. It should be noted that this specific experiment was intended for qualitative trend validation of the LSPR-active platform’s response to rapid pressure fluctuations. While concurrent gold-standard tonometry was not performed to minimize mechanical interference with the lens–cornea interface, the observed spectral shifts are consistent with the ex vivo physical mapping model established in our porcine eye studies, which demonstrated reliable sensitivity between 10–50 mmHg.

The acute high-IOP model eyes in groups A–E were compared and studied. Corneal tissue has distinct layers, with neatly arranged epithelial cells and a regular and parallel arrangement of collagen plates in the stromal layer. No obvious edema is observed. Corneal endothelial cells have round pale nuclei with unclear cell boundaries, and no obvious edema or vacuolar changes are observed. Compared with the pathological sections of corneal tissue from experimental rabbits wearing static clothing during the same period, no significant changes were observed. The arrangement of fibrous scaffolds in the trabecular tissue is regular, and the morphology of trabecular cells is normal. The pore structure inside the small beam mesh is clear. No inflammatory cell infiltration, fibrosis, proliferation, or neovascularization was observed. Compared with the pathological sections of trabecular meshwork tissue in experimental rabbits wearing static clothing during the same period, no significant changes were observed.

## 5. Discussion

Our study proposes a smart contact lens based on the LSPR principle for non-invasive, continuous IOP monitoring. By embedding AuNPs into the periphery of a silicone hydrogel lens, the system leverages IOP-induced changes in corneal curvature to modulate the interparticle spacing of the AuNPs. SEM characterization reveals that while the overall nanoparticle density (5–8 particles/µm^2^) remains low to maintain optical transparency, the presence of locally coupled domains is critical for signal transduction. Although the non-uniformity in particle size (30–50 nm) results in an ensemble-averaged spectral response, the optical signal is dominated by these regions of high field enhancement, enabling highly sensitive detection of microscopic tensile deformations.

The transduction of IOP fluctuations into LSPR signals is governed by the biomechanical interaction between the cornea and the hydrogel matrix. As IOP increases, the expansion of the corneal curvature exerts circumferential tensile strain on the lens periphery, which is translated into a microscopic increase in the interparticle spacing (d) within the embedded AuNP clusters, manifesting as a detectable blue shift in the extinction spectrum. Conversely, the excellent elastic recovery of the silicone hydrogel facilitates the reduction of interparticle distances as IOP returns to baseline, resulting in a spectral red shift. To further validate this mechanism, we evaluated the potential contribution of refractive index (RI) alterations during hydrogel stretching. Given that the Young’s modulus of the silicone hydrogel is approximately 0.3 MPa, the strain induced by a maximum IOP of 50 mmHg (≈6.7 kPa) is limited to approximately 2% [34,35]. Within this small elastic deformation range, the contribution of RI fluctuations is negligible compared to the near-field coupling modulation within the investigated strain regime. Therefore, the observed LSPR shifts are primarily governed by the modulation of interparticle spacing, providing the primary physical mechanism for tracking dynamic IOP fluctuations within the 10–50 mmHg range.

Our study successfully validates the fundamental physical transduction mechanism of the LSPR-active silicone hydrogel matrix through a combination of ex vivo porcine and in vivo rabbit models. In the experiment, we observed that with the increase and decrease in IOP, the extinction spectrum peak of the corneal contact lens showed regular left and right shifts, which is consistent with theoretical predictions. In this study, ex vivo pig eyeball anterior chamber catheterization was used to control IOP. It was observed that as IOP increased, the corneal curvature radius increased, and the fitting curve between IOP and curvature radius was approximately proportional to the slope. This is similar to the results of Lam et al.’s study on human eyes [9]. By integrating a pre-wear corneal curvature radius measurement into the sensor’s algorithm, the influence of corneal geometry on the stress–strain conversion factor can be precisely compensated, ensuring that the signal remains robust and resolvable. Our physical mapping model demonstrated a response sensitivity of −0.12 nm/mmHg, confirming that the AuNP–silicone hydrogel system can reliably translate dynamic ocular strain into predictable spectral shifts. It is important to acknowledge that the current study validates the LSPR sensing mechanism primarily through a macroscopic, functionally driven approach. The ex vivo porcine eye model functioned as a physiologically relevant controlled strain assay, establishing the correlation between macroscopic ocular deformation and optical spectral shifts (−0.12 nm/mmHg). However, direct nanoscale experimental validation of interparticle spacing variations under planar biaxial stretching, as well as rigorous electrodynamic simulations (e.g., finite difference time domain methods), were not conducted in this work. The transduction principle relies on the well-documented physical consensus regarding strain-modulated plasmonic coupling in elastomeric matrices. Future studies integrating multi-physics computational modeling will be required to quantitatively elucidate the precise local electromagnetic field variations within the 3D hydrogel network during micro-strain.

It is important to note that the “swelling-induced nano-doping” technique integrates AuNPs within the complex 3D polymeric network of the silicone hydrogel, rather than merely on a 2D surface. While standard 2D SEM characterization confirms the successful embedding and general meso-scale distribution of the nanoparticles, it inherently cannot fully capture the critical out-of-plane (Z-axis) interparticle distances responsible for volumetric plasmonic coupling. Consequently, the observed LSPR spectral shifts represent an ensemble-averaged optical response from numerous 3D high-density nanoclusters undergoing macroscopic mechanical strain. Future fundamental studies utilizing 3D tomographic imaging techniques (e.g., Cryo-TEM) are warranted to precisely quantify the 3D spatial geometry of these nanoclusters. Nevertheless, the robust linear sensitivity (−0.12 nm/mmHg) observed in our ex vivo and in vivo functional assessments validates the reliability of this ensemble transduction mechanism for macroscopic IOP monitoring.

The ultrafast nature of LSPR collective oscillation—typically occurring on femtosecond to picosecond timescales—ensures that the optical response of the functionalized lens is effectively instantaneous relative to physiological IOP fluctuations [36]. By embedding AuNPs within this high-sensitivity zone, we maximized optical transduction efficiency without requiring internal power supplies. The system demonstrated measurable optical response trends (R^2^ = 0.859 in fresh biological specimens) within the physiological range of 10–50 mmHg. While active electronic systems such as the wireless PT-symmetric sensors by Xiao et al. may offer higher raw sensitivity, our platform provides a passive, chipless alternative that maintains critical clinical parameters, including optical transparency and high oxygen permeability [37].

During the 21-day rabbit study, the lenses demonstrated high centration stability, with the functionalized peripheral zone consistently aligned with the limbal area. This stability is crucial, as significant decentration could lead to optical path misalignment and subsequent signal noise. We observed a small physiological movement which is consistent with ideal clinical fitting criteria. This movement not only facilitates tear exchange and maintains corneal oxygenation but also confirms that the lens-cornea interface remains dynamic yet stable enough for reliable signal acquisition.

Furthermore, a key challenge for wearable optical sensors is the potential for biofouling (e.g., protein and lipid deposition) to obstruct the visual axis or interfere with the LSPR signal. Our findings show that the central optical zone remained transparent throughout the longitudinal study, with no significant mucoid plaque or opacification observed. This suggests that our “peripheral doping” strategy, combined with the inherently low-fouling nature of the silicone hydrogel matrix, effectively preserves visual clarity while isolating the sensing units from the central field of view. These observations provide strong evidence that the LSPR-active system can maintain both its structural integrity and diagnostic functionality in a complex biological environment. The complex ocular environment presents challenges for the long-term stability of LSPR-based sensors. The non-specific adsorption of tear film components, such as lysozyme, albumin, and lipids, onto the lens surface or within the hydrogel matrix can alter the local refractive index surrounding the AuNPs, potentially leading to baseline spectral shifts. While our current architecture leverages the silicone hydrogel network as a physical barrier to shield the embedded AuNPs, biofouling remains a factor that could influence sensitivity over extended wear. To mitigate this, future iterations will explore anti-fouling strategies, including the surface grafting of polyethylene glycol (PEG) or zwitterionic polymers onto the lens surface to minimize protein deposition and enhance the signal-to-noise ratio for reliable long-term IOP monitoring.

Hajrasouliha et al. recently established that gold nanoparticle formulations are biocompatible for intraocular use, resulting in no significant cell death or retinal structural damage [38]. This study evaluated the ocular surface safety of long-term wearing of AuNP silicone hydrogel contact lenses in live rabbits. Clinical indicators, including tear secretion, corneal fluorescein staining, and inflammation index, all remained within normal ranges, showing no signs of dry eye, epithelial damage, or significant inflammation. Pathological examination revealed mild conjunctival vasodilation and minimal inflammatory cell infiltration, consistent with known physiological responses to contact lens wear and considered within acceptable limits [39,40]. Furthermore, PAS staining showed no significant difference in conjunctival goblet cell count among groups, indicating maintained ocular surface defense. HE staining of the cornea and trabecular meshwork revealed no notable morphological abnormalities or inflammatory infiltration. The absence of Ki67-positive cells suggested no pathological proliferation, and stable corneal endothelial cell counts confirmed the absence of chronic hypoxia. The multi-dimensional assessment demonstrates good ocular surface biocompatibility and supports the long-term wearing safety of the tested lens, providing valuable experimental evidence for its clinical application. Longitudinal studies over 21 days have confirmed the safety of gold nanoparticles in animal models, showing none or negligible toxicity while maintaining anterior segment tolerance [41].

In the acute ocular hypertension model, the LSPR sensor tracked dynamic IOP fluctuations induced by repeated intravitreal injections, exhibiting immediate spectral blue shifts corresponding to transient pressure spikes. Upon each injection, the extinction spectrum exhibited an immediate and sharp blue shift, effectively capturing the transient pressure spikes. This was followed by a progressive red shift back toward the baseline, reflecting the sensor’s ability to monitor the eye’s natural pressure recovery process. While the magnitude and rate of these shifts varied slightly across consecutive injections—likely due to the dynamic intraocular volume changes—the sensor consistently provided continuous optical tracking of the IOP’s ”rise-and-fall” patterns. These results, complemented by the observed rapid response following mannitol administration, confirm that the LSPR-active platform can reliably track complex, multi-phasic pressure trends in a living biological system.

The population undergoing IOP monitoring may experience a pathological state of elevated IOP; therefore, it is necessary to evaluate the tolerance of short-term high IOP in the anterior segment tissue after continuous wearing of contact lenses to reduce the possibility of iatrogenic damage. After wearing contact lenses continuously for a long time, even under short-term high IOP, the corneal tissue and trabecular meshwork tissue maintain a relatively stable structure. There was no infiltration of blood vessels, lymphatic vessels, or inflammatory cells in the corneal tissue, indicating that the wearing of contact lenses did not have a significant pathological effect on the corneal tissue. At the same time, the arrangement of fibrous scaffolds in the trabecular tissue was regular, the morphology of trabecular cells was normal, the pore structure was clear, and no pathological changes such as inflammatory cell infiltration, fibrosis, proliferation, or neovascularization were observed. These observation results did not show significant differences compared to the histopathological sections of the tissues of rabbits wearing static clothing during the same period. This result suggests that the wearing of corneal contact lenses can maintain relative stability in the anterior segment tissue of live rabbit eyes that have undergone transient high IOP for a long time, and have certain adaptability and tolerance to high IOP, providing more detailed and scientific basis for the clinical application of corneal contact lenses.

Although this study has confirmed that AuNP–silicone hydrogel contact lenses have certain advantages in IOP monitoring, ocular surface safety, and high-IOP tolerance, there are still some issues that need to be addressed. Current research is mainly based on animal experiments, and there are certain differences in physiological structure and function between animal models and human eyes. Future human clinical trials are needed to further verify its accuracy, safety, and effectiveness in humans.

While this study focuses on the targeted spectral range (450–750 nm) to capture the primary LSPR shifts associated with IOP fluctuations, we acknowledge that a full-spectrum UV-vis characterization under static conditions would provide additional insights into the optical baseline and secondary electronic transitions of the hybrid system. However, standard full-spectrum scanning instruments typically require longer acquisition times. To achieve the necessary high temporal resolution (0.2 s per acquisition) for real-time, dynamic in vivo IOP monitoring, our experimental protocol utilized a fiber optic spectrometer focused on the visible window. This methodological choice ensured continuous 5 Hz data collection, successfully capturing the transient functional LSPR peak shifts without compromising the temporal accuracy required for dynamic tracking. Future work will involve broad-band spectroscopic analysis to further refine the dielectric modeling of the AuNP–hydrogel interface and to explore the potential for multi-wavelength error correction in diverse lighting environments.

In this proof-of-concept study, we relied on the correlation between spectral displacement and established ex vivo calibration models to infer IOP changes, demonstrating a fundamental engineering pathway for passive wearable diagnostics. However, as the current study prioritized evaluating the histopathological tolerance of anterior segment tissues under acute hypertensive stress, simultaneous anterior chamber paracentesis was deliberately avoided to preserve the structural integrity of the globe and prevent iatrogenic interference with pathological findings. To bridge this gap, our ongoing research involves synchronous cross-validation with clinical tonometry in a larger cohort, where a multivariate correction model, integrating central corneal thickness and corneal hysteresis, is being developed to refine the sensor’s absolute accuracy and ensure diagnostic reliability across diverse physiological conditions.

At the current experimental stage, spectral detection mainly relies on fiber optic spectrometers. For later clinical translation applications, an integrated optical acquisition module and signal processing unit can be designed and miniaturized to be embedded within the contact lenses. Integrating this detection system into the smart contact lens framework still faces multifaceted technical challenges, primarily manifested in the following aspects: Micro-light sources in wearable devices require excellent stability, which can be enhanced through strategies such as constant current driving, power management, and temperature compensation to improve the stability of light source output. Motion artifacts arising from eye blinking or head movements must be suppressed using advanced signal processing algorithms, such as adaptive filtering or motion-triggered data gating. By optimizing the optical structure design, a relatively fixed geometric relationship is established between the lens and the detection assembly, thereby reducing positional deviations during wear and achieving consistent optical alignment between the lens periphery and the frame-mounted detector. This study provides a preliminary foundation for achieving wearable and continuous optical detection, with related integration solutions to be further explored and refined in subsequent work.

Stable spatial alignment enables optical reading of the sensing area of contact lenses, and data can be transmitted to mobile terminals (e.g., smartphone apps) via wireless methods such as Bluetooth for analysis and display, achieving portable detection. On the other hand, although the contact lens performs well in IOP fluctuation monitoring, its precision in IOP measurement and long-term stability need further improvement. Current monitoring results may be affected by various factors, such as tear composition and the wearing state of the contact lens, necessitating the enhancement of the stability and precision of monitoring technology to ensure reliable IOP data for clinical use.

## 6. Conclusions

This study successfully developed an LSPR-active silicone hydrogel contact lens for non-invasive monitoring of dynamic ocular strain associated with IOP fluctuations. By leveraging the synergistic effect of AuNPs’ plasmonic properties and the hydrogel’s elasticity, the sensor effectively transduces physiological micro-strain into predictable spectral shifts with a moderate linear correlation (R^2^ = 0.859). While ex vivo porcine models validated the fundamental transduction mechanism and robust correlation within physiological ranges, the 21-day in vivo rabbit study confirmed that the device maintained ocular surface parameters within normal physiological limits (*p* > 0.05 for histological comparisons) and structural stability. These findings establish a promising technical foundation for glaucoma management. Future clinical translation will focus on integrating individualized calibration protocols and AI-enhanced algorithms to account for person-specific corneal biomechanics, ensuring the precision required for human diagnostics.

## Data Availability

The original contributions presented in this study are included in the article. Further inquiries can be directed to the corresponding authors.
